# 高效液相色谱-串联质谱法快速同时测定土壤中草甘膦、草铵膦及其代谢物

**DOI:** 10.3724/SP.J.1123.2021.08005

**Published:** 2022-03-08

**Authors:** Hua PING, Fang ZHAO, Cheng LI, Beihong WANG, Hongling KONG, Yang LI, Zhihong MA

**Affiliations:** 1.北京市农林科学院北京农业质量标准与检测技术研究中心, 北京 100097; 1. Beijing Research Center for Agricultural Standards and Testing, Beijing Academy of Agriculture and Forestry Sciences, Beijing 100097, China; 2.农业农村部农产品质量安全风险评估实验室(北京), 北京 100097; 2. Risk Assessment Laboratory for Agro-products Quality and Safety (Beijing), Ministry of Agriculture and Rural Affairs, Beijing 100097, China

**Keywords:** 高效液相色谱-串联质谱, 草甘膦, 草铵膦, 代谢物, 土壤, 非衍生化, high performance liquid chromatography-tandem mass spectrometry (HPLC-MS/MS), glyphosate, glufosinate, metabolites, soil, non-derivatization

## Abstract

建立了快速同时测定土壤中草甘膦(GLY)、草铵膦(GLUF)及其代谢物的高效液相色谱-串联质谱(HPLC-MS/MS)分析方法。分别对前处理和色谱-质谱条件进行优化,样品采用0.5 mol/L氨水作为溶剂振荡提取,离心,上清液过滤膜后,直接采用HPLC-MS/MS测定,电喷雾离子源(ESI^-^),多反应监测(MRM)模式下检测,外标法定量。结果表明,采用Dikma Polyamino HILIC色谱柱(150 mm×2.0 mm, 5 μm)进行分离,目标化合物峰形良好,响应值高。GLY及其代谢物氨甲基膦酸(AMPA)在5.0~500 μg/L范围内线性关系良好;GLUF及其代谢物*N*-乙酰基-草铵膦(NAG)、3-(甲基膦基)丙酸(MPP)在2.0~500 μg/L范围内线性关系良好;5种化合物的相关系数(*r*^2^)均大于0.998,可以满足定量分析的要求。GLY、GLUF及其代谢物的方法检出限(LOD)在2.0~4.0 μg/kg之间,方法定量限(LOQ)在6.7~13.3 μg/kg之间。在空白土壤样品中添加0.02、0.05、0.2 mg/kg 3个水平的5种化合物混合标准溶液考察回收率,结果表明,低有机质含量土壤中5种化合物的平均加标回收率在74.2%~101%之间,相对标准偏差(RSD)在0.93%~6.8%之间;高有机质含量土壤中5种化合物的平均加标回收率在90.8%~116%之间,RSD在0.40%~7.1%之间。采用建立的方法对实际土壤样品进行测定,AMPA、GLY、MPP、GLUF、NAG的检出率分别为45%、25%、10%、5%和5%,最大残留量分别为147、35.2、154、21.6和11.0 μg/kg,说明土壤中GLY、GLUF及其代谢物残留需要引起一定的关注。该方法简化了前处理步骤,具有简单、快速、绿色环保、成本低、准确度和灵敏度高、重现性好等优点,适用于大批量不同有机质含量土壤样品的检测,为研究其在土壤中的残留状况和环境行为提供了可靠的技术支持。

## Abstract (en)

Glyphosate (GLY) and glufosinate (GLUF) are non-selective translocated herbicides that are used in agricultural and non-agricultural land worldwide. The extensive use of GLY and GLUF may lead to their accumulation in soil, which causes soil pollution and affects the soil micro-ecological environment; the accumulated GLY and GLUF also migrate to groundwater via leaching. However, GLY, GLUF, and their metabolites are highly water-soluble and lack chromogenic and fluorescent groups, making them difficult to analyze. Currently, derivatization methods are mostly used to detect GLY, GLUF, and their metabolites. However, these methods also have some drawbacks, such as complex operation, long time consumption, and poor stability. In addition, these compounds are easily passivated and made inactive in soil; they also react with organic matter, humic acid, metal oxides, and heavy metal ions, making their extraction from soil difficult. To date, the method for the determination of GLY, GLUF, and their metabolites in soil is limited. Therefore, it is necessary to establish a quick and sensitive method to determine the residues of GLY, GLUF, and their metabolites in soil.

In this study, a high performance liquid chromatography-tandem mass spectrometry (HPLC-MS/MS) method was developed for the determination of GLY, GLUF, and their metabolites in soil. Parameters like extraction solvent, extraction temperature, extraction time, and adsorbents, which affected the extraction efficiencies, were optimized. Finally, the soil samples were extracted with 0.5 mol/L ammonia solution in a bath shaker at 50 ℃, and then centrifuged at 10000 r/min for 5 min. The supernatant was filtered through 0.2-μm syringe filters and then determined by HPLC-MS/MS. A Dikma Polyamino HILIC column (150 mm×2.0 mm, 5 μm) was used for chromatographic separation with good peak shape and high response of the target compounds. Ammonium acetate (0.2 mmol/L) with 0.07% ammonia aqueous solution and acetonitrile were used as the mobile phase. The flow rate of the elute was 0.25 mL/min. MS/MS was conducted under multiple reaction monitoring (MRM) mode using an electrospray ionization (ESI) source, and was quantified by the external standard method using matrix-matched calibration curves. All the target compounds were ionized in the negative ionization mode. The linear ranges of GLY and its metabolites were between 5.0 and 500 μg/L, and those of GLUF and its metabolites were between 2.0 and 500 μg/L. Linear correlation coefficients were greater than 0.99. The limit of detection (LOD) and limit of quantification (LOQ) were assessed using signal-to-noise (*S/N*) ratios of 3 and 10, respectively. The LOD and LOQ values of both GLY and (aminomethyl)phosphonic acid (AMPA) were 4.0 and 13.3 μg/kg, respectively. The LOD and LOQ values of GLUF, MPP, and *N*-acetyl glufosinate (NAG) were 2.0 and 6.7 μg/kg, respectively. Method accuracy was acquired by recovery test at three spiked levels (0.02, 0.05, 0.2 mg/kg). The average recoveries of five targets spiked in soil with low organic matter content were 74.2%-101%, and the relative standard deviation (RSD) was 0.93%-6.8%; the average recoveries of the five targets spiked in soil with high organic matter content were 90.8%-116%, and the RSD was 0.40%-7.1%. The established method was used to determine 20 soil samples in peach orchard, and the detection rates of AMPA, GLY, MPP, GLUF and NAG were 45%, 25%, 10%, 5% and 5%, respectively. The maximum residues were 147, 35.2, 154, 21.6 and 11.0 μg/kg, respectively. This method is simple, rapid, green, inexpensive, allows pretreatment without organic reagents, and affords high accuracy, high sensitivity, and good reproducibility. The method is suitable for testing a large number of soil samples with different organic matter contents. It can provide reliable technical support for the study of residue status and environmental behavior in soil.

草甘膦(GLY)和草铵膦(GLUF)均为灭生性除草剂,在世界范围内广泛用于农业及非农业用地除草^[1]^。GLY和GLUF的大量使用在给农业增产的同时,势必会在土壤中累积,造成土壤污染,影响土壤微生态环境,还会通过淋溶迁移到地下水中^[2,3,4]^。Silva等^[5]^对欧盟成员国300份土壤样品进行监测发现,45%的样品中检出了GLY和氨甲基膦酸(AMPA)残留。近年来,仅有少数学者对国内土壤中GLY和GLUF进行了监测,也检出了GLY、AMPA及GLUF残留^[6,7,8]^,这与土壤中GLY、GLUF及其代谢物检测困难有一定的关系。

由于GLY、GLUF及其代谢物均为强极性化合物,缺少发色和荧光基团,采用常规的检测技术对其进行定性定量分析难度较大^[6,9]^。目前,文献中报道的GLY、GLUF及其代谢物的测定方法主要有气相色谱法^[10]^、液相色谱法^[11]^、气相色谱-质谱联用法^[12]^、液相色谱-质谱联用法^[13]^、离子色谱法^[14,15]^、毛细管电泳法^[16]^等,涉及的检测对象以植物源性食品或者水样居多。关于土壤中的检测方法相对较少,目前国内仅有一项关于土壤中GLY的检测标准^[17]^。这是由于土壤中的有机质、腐殖酸、金属氧化物及重金属离子等对GLY、GLUF及其代谢物有很强的吸附和络合作用,导致其在土壤中提取困难^[3,7,18,19]^。文献及标准中土壤中GLY、GLUF检测前处理多采用衍生化方法,但是衍生化方法存在步骤繁琐、耗时长、重复性差、须严格控制操作条件、对分析人员要求高等问题^[3,11,20]^。与衍生化方法相比,非衍生化方法具有操作简便、重复性好的优点。近年来,孙文闪等^[7]^和周芹等^[21]^分别采用水-二氯甲烷、磷酸钠-柠檬酸三钠作为提取溶剂,非衍生化-液相色谱-质谱联用方法测定土壤中GLY及其代谢物AMPA和GLUF等3种化合物,但是这两种方法均需要过固相萃取小柱净化,步骤繁琐,且成本较高。目前关于土壤中GLY、AMPA、GLUF及其代谢物*N*-乙酰基-草铵膦(NAG)、3-(甲基膦基)丙酸(MPP)等5种化合物同时测定的方法尚未见报道,因此有必要建立一套实施方便、试验成本低、灵敏度高、重复性好的快速同时测定土壤中GLY、GLUF及其代谢物等5种化合物的检测技术。

本文采用氨水作为提取溶剂,无须过固相萃取小柱净化,无须衍生化处理,以HILIC色谱柱为分离柱,实现了不同有机质含量土壤中GLY、AMPA、GLUF、MPP和NAG等5种化合物的高效液相色谱-串联质谱法快速同时测定。该方法具有简单、快速、前处理无须有机试剂、绿色环保、成本低、准确度和灵敏度高、重复性好等优点,可以满足不同有机质含量土壤样品的批量检测,为研究其在土壤中的残留状况和环境行为提供可靠的技术支持。

## 1 实验部分

### 1.1 仪器与试剂

ACQUITY超高效液相色谱仪(美国Waters公司); Xevo TQ-S三重四极杆质谱仪,配有电喷雾电离(ESI)源(Waters公司); 3K30高速冷冻离心机(美国Sigma公司); Classic UF纯水机(美国Pall公司);水浴恒温振荡器(上海旻泉仪器有限公司); 0.2 μm微孔过滤膜(美国Gelman Laboratory公司);乙腈(HPLC级,美国Fisher公司);氨水(色谱纯,上海阿拉丁试剂公司);十八烷基键合硅胶(C_18_)、*N*-丙基乙二胺(PSA)(天津博纳艾杰尔科技有限公司)。

GLY(纯度98.6%)、GLUF(纯度99.2%)、AMPA(纯度99.0%)、NAG(纯度97.4%)、MPP(质量浓度100 mg/L)购自德国Dr. Ehrenstorfer公司。将5种除草剂标准品分别用纯水配成10.0 mg/L标准储备液,实验时用土壤空白基质溶液配成不同质量浓度的工作液。

方法学考察试验用空白土壤样品采自北京市顺义区某桃园,低有机质含量样品(有机质含量为2.89%, pH值为7.0,阳离子交换量为12.3 cmol/kg),高有机质含量样品(有机质含量为7.38%, pH值为7.1,阳离子交换量为13.0 cmol/kg)。

### 1.2 样品前处理方法

称取土壤5 g(过60目筛)于50 mL离心管中,加入0.5 mol/L氨水溶液20 mL,涡旋混匀,置于水浴恒温振荡器中于50 ℃加热振荡提取60 min,在4 ℃下10000 r/min离心5 min,取上清液过0.2 μm滤膜,HPLC-MS/MS测定。

土壤空白基质溶液制备:选取空白土壤(不含目标化合物的土壤)按照上述前处理方法进行提取后,离心,取上清液过0.2 μm滤膜,即得。

### 1.3 分析方法

1.3.1 高效液相色谱条件

色谱柱:Dikma Polyamino HILIC(150 mm×2.0 mm, 5 μm);色谱柱温:40 ℃;样品室温度:10 ℃;进样体积:10 μL;流动相A:水相(0.2 mmol/L乙酸铵+0.07%氨水溶液),流动相B:乙腈,流速为0.25 mL/min;梯度洗脱条件:0~1.0 min, 25%A; 1.0~2.0 min, 25%A~95%A; 2.0~9.0 min, 95%A。

1.3.2 质谱条件

离子源:ESI源;扫描方式:负离子模式;监测模式:多反应监测(MRM)模式;毛细管电压:2.5 kV;雾化气温度:500 ℃;去溶剂气流量:1000 L/h;碰撞气流量:0.20 mL/min;离子源温度:150 ℃。

## 2 结果与讨论

### 2.1 HPLC-MS/MS条件优化

分别对5种化合物的质谱条件进行了优化,包括锥孔电压、碰撞能量、毛细管电压等,使每种化合物的母离子与特征碎片离子强度达到最大,将响应值最大的碎片离子设定为定量离子,次级响应离子为定性离子,优化后的质谱参数见表1。

**表1:** 5种化合物的质谱分析参数

No.	Compound	Compound abbr.	Retention time/min	Parent ion (m/z)	Daughter ions (m/z)	Cone voltage/V	Collision energies/eV
1	glyphosate	GLY	7.73	168.05	63.11^*^, 150.00	20	20, 10
2	(aminomethyl)phosphonic acid	AMPA	4.82	110.04	63.11^*^, 79.18	60	18, 20
3	3-(methylphosphinico)propionic acid	MPP	6.65	151.06	63.14, 107.00^*^	46	24, 20
4	glufosinate	GLUF	6.22	180.11	85.10^*^, 95.05	50	20, 18
5	N-acetyl glufosinate	NAG	6.65	222.09	134.20, 136.12^*^	50	24, 22

* Quantitative ion.

GLY和GLUF及其代谢物极性均较强,在常规反相色谱柱上保留较小,因此应选择亲水色谱柱进行分离。实验中分别比较了Restek Polar X离子色谱柱(100 mm×2.1 mm, 2.7 μm)、ACQUITY UPLC BEH Amide色谱柱(100 mm×2.1 mm, 1.7 μm)和Dikma Polyamino HILIC色谱柱(150 mm×2.0 mm, 5 μm)对5种化合物色谱峰形、响应值、出峰时间的影响。结果表明,GLY、GLUF及其代谢物在HILIC色谱柱上峰形良好,且响应值高,因此最终选择了HILIC色谱柱。

考察了流动相、柱温、流速及梯度洗脱条件对目标化合物峰形、响应值和离子化效率的影响。其中,流动相组成是一个重要的影响因素,分别考察了水相中加入不同浓度乙酸铵及不同比例氨水对色谱峰形及响应值的影响。结果表明,水相中加入0.2 mmol/L乙酸铵和0.07%氨水时有助于目标化合物离子化,响应值最高。最终优化后的流动相条件为水相(0.2 mmol/L乙酸铵+0.07%氨水溶液)和乙腈。各个化合物在MRM模式下的色谱图见图1。

**图1:**
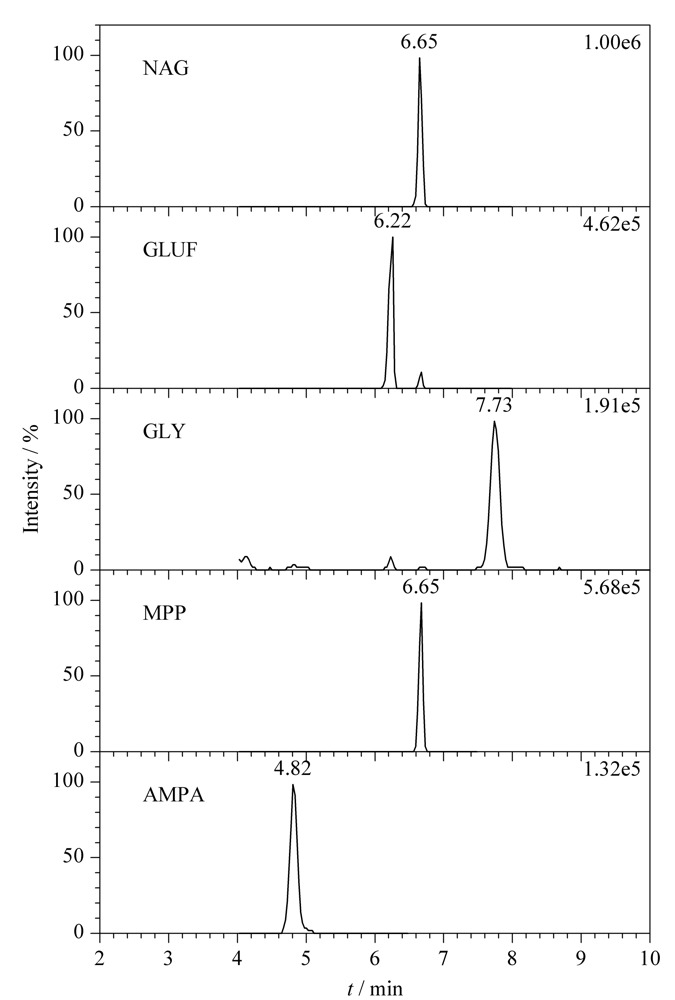
5种化合物在MRM模式下的色谱图

### 2.2 前处理条件的优化

2.2.1 提取溶剂的优化

考察了不同提取溶剂对GLY、GLUF及其代谢物提取效率的影响。分别选择纯水、水-乙腈(1:1, v/v)、柠檬酸盐缓冲溶液(4%柠檬酸钠、2%柠檬酸氢二钠)、氨水作为溶剂,结果表明,氨水提取和柠檬酸盐提取回收率无明显差别,其余溶剂提取回收率均较低。这是由于GLY、GLUF及其代谢物为酸性,采用碱性溶液作为提取溶剂,有利于将被土壤有机质结合的GLY、GLUF及其代谢物变成离子形态,从土壤中游离出来,可以有效降低土壤对目标化合物的吸附,从而提高回收率。文献^[21,22]^采用磷酸钠、柠檬酸三钠作为提取溶剂,发现随着提取液pH的增加,土壤对GLY和AMPA的吸附量减少,提取效率变高,此结果与前人研究结果相一致。考虑到柠檬酸盐为不挥发性盐,进入质谱后会在离子源部分结晶、沉积,影响目标化合物电离,需要经常清洗和保养。由于氨水具有挥发性,可以直接进入质谱,省去转换溶剂的步骤,因此选用氨水作为提取溶剂。考察了不同浓度氨水溶液(0.05、0.1、0.2、0.5、1、2、3 mol/L氨水)对GLY、GLUF及其代谢物色谱峰形、响应值和回收率的影响(见图2)。结果表明,采用0.5 mol/L和1.0 mol/L氨水溶液提取5种化合物,回收率均在70%~120%之间,从节省试剂的角度出发,最终选择0.5 mol/L氨水溶液作为提取溶剂;在该条件下,5种目标化合物在质谱中的离子化效率高,响应值高,而且峰形好。

**图2:**
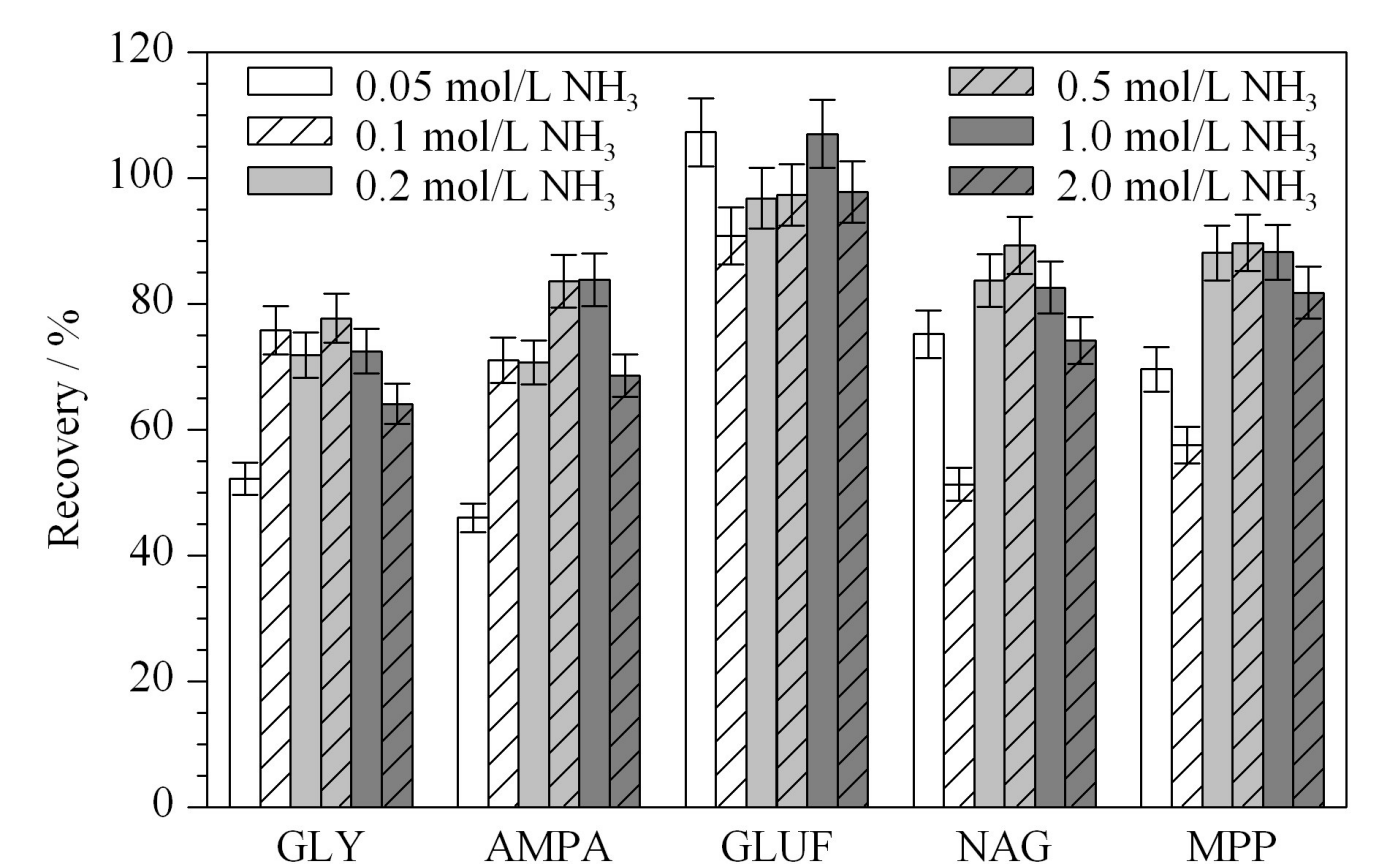
氨水浓度对GLY、GLUF及其代谢物回收率的影响

2.2.2 净化方法的优化

目前常用的净化方法有分散固相萃取法和固相萃取法。C_18_和PSA是农药残留净化中常用的分散固相萃取吸附剂,分别考察了这两种吸附剂对5种化合物回收率的影响。C_18_主要用于吸附脂类和甾醇类等非极性化合物,实验发现C_18_净化和未净化无明显差别,这说明提取液中的疏水性基质对目标化合物的检测无明显影响。PSA净化后回收率低,这是由于目标化合物含有羧基、膦酸基等基团,PSA在吸附提取液中极性色素、有机酸、脂肪酸等杂质的同时,对目标化合物也产生了吸附^[6,23]^。固相萃取法中常将HLB固相萃取小柱用于GLY等极性化合物检测的样品净化,可以有效地去除色素、脂肪及蛋白质等杂质^[7,21]^。对于提取液过HLB固相萃取小柱和不过小柱做了对比试验,发现回收率无明显差异;过HLB柱后,GLUF和NAG的基质效应反而增强;而且采用固相萃取柱净化成本高,操作复杂,不适用于大批量样品检测。为节省成本,满足大批量样品快速测定的需要,因此最终选择不使用吸附剂和固相萃取小柱进行净化。

2.2.3 提取温度的优化

温度也是影响提取效率的一个因素,该实验对25、40、50、60、70 ℃ 5个不同提取温度进行了考察,结果见图3。除NAG随着提取温度升高回收率不断提高以外,其他几种化合物回收率受温度影响不太明显。综合考虑,优化后的提取温度为50 ℃。

**图3:**
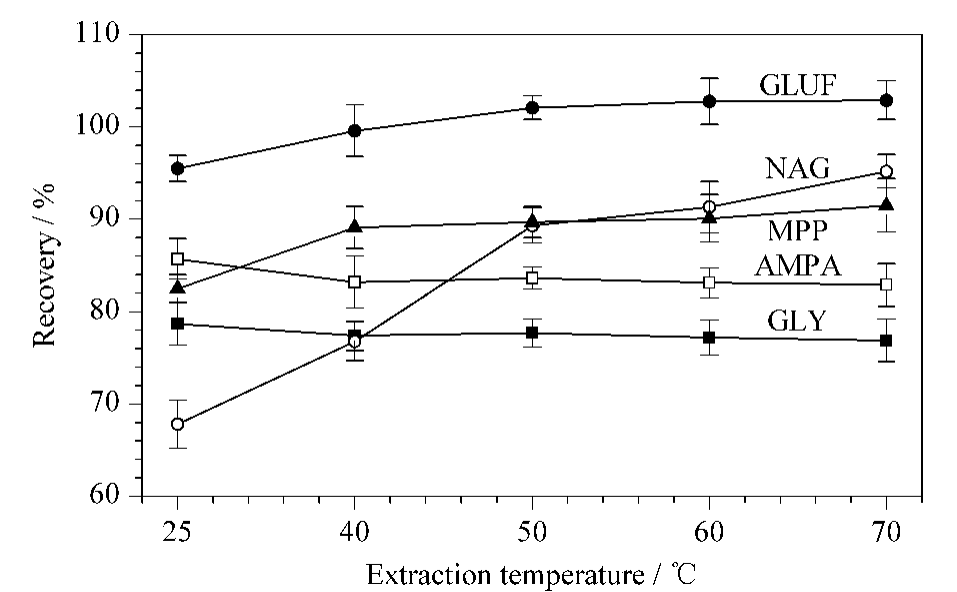
提取温度对GLY、GLUF及其代谢物回收率的影响(*n*=3)

2.2.4 提取次数的优化

实验考察了提取1次和提取2次对5种化合物回收率的影响,发现提取次数对回收率无明显影响,因此从提高效率和节约成本的角度考虑,最终选择提取次数为1次。

### 2.3 基质效应(ME)

基质效应可能会对目标化合物的离子化效率、响应值造成影响,这是由于样品基质中存在的其他干扰成分在离子化过程中与目标化合物竞争,表现为增强或者抑制效应,如果基质效应强可能会导致目标化合物的定量结果不准确^[24]^。基质效应计算方法见公式(1)^[25,26]^:


(1) ME=\left(\frac{A_{m}}{A_{s}}-1 \right)\times 100\%

其中,*A_m_*为土壤基质溶液配制的标准溶液峰面积,*A_s_*为纯溶剂配制的标准溶液峰面积。|ME|≤20%,表明无明显基质效应;20%<|ME|≤50%,表明基质效应中等;|ME|>50%,表明对目标化合物有较强的基质效应。实验中比较了0.05 mg/L溶剂标准溶液和土壤基质标准溶液的峰面积,平行测定6次。结果表明,GLY、AMPA、GLUF、MPP、NAG基质效应分别为33.9%、31.0%、8.6%、21.4%、4.7%, GLY、AMPA和MPP表现为中等基质效应,GLUF和NAG无明显基质效应。综合考虑,本实验中选用基质配制标准溶液的方式来进行定量分析,以降低基质效应的影响。

### 2.4 方法学考察

2.4.1 线性范围与检出限

配制2.0、5.0、10、50、100、200、500 μg/L的土壤基质混合标准溶液,以质量浓度(*x*, μg/L)为横坐标,峰面积(*y*)为纵坐标,绘制基质标准工作曲线(见表2)。结果表明,GLY及其代谢物在5.0~500 μg/L范围内线性关系良好,GLUF及其代谢物在2.0~500 μg/L范围内线性关系良好,相关系数(*r*^2^)均大于0.99,可以满足定量分析的要求。以3倍信噪比计算,GLY及其代谢物的方法检出限(LOD)为4.0 μg/kg,GLUF及其代谢物的LOD值为2.0 μg/kg;以10倍信噪比计算,GLY及其代谢物的方法定量限(LOQ)为13.3 μg/kg,GLUF及其代谢物的LOQ值为6.7 μg/kg(见表2)。与标准方法相比^[17]^,该方法GLY的线性范围较宽、检出限较低。

**表2:** 5种化合物的线性范围、线性方程、相关系数、方法检出限和定量限

Compound	Linear range/ (μg/L)	Linear equation	r^2^	LOD/ (μg/kg)	LOQ/ (μg/kg)
GLY	5.0-500	y=158.5x+488.5	0.9980	4.0	13.3
AMPA	5.0-500	y=60.3x+94.0	0.9994	4.0	13.3
MPP	2.0-500	y=144.8x+108.7	0.9986	2.0	6.7
GLUF	2.0-500	y=114.4x+153.1	0.9988	2.0	6.7
NAG	2.0-500	y=315.1x+580.4	0.9993	2.0	6.7

*y*: peak area; *x*: mass concentration, μg/L; LOD: limit of detection; LOQ: limit of quantification.

2.4.2 精密度和准确度

由于土壤有机质含量是影响GLY、GLUF及其代谢物吸附的主要因素,分别对低有机质含量(有机质含量在5%以下)和高有机质含量(有机质含量在5%~10%之间)的土壤样品按照上述优化的实验方法进行加标回收试验。分别在不同有机质含量的空白土壤样品中添加0.02、0.05、0.2 mg/kg 3个水平的5种除草剂的混合标准溶液,每个添加水平重复测定6次,计算平均回收率和相对标准偏差(RSD)。结果表明,低有机质含量土壤中5种化合物的平均加标回收率在74.2%~101%之间,RSD在0.93%~6.8%之间(见表3);高有机质含量土壤中5种化合物的平均加标回收率在90.8%~116%之间,RSD在0.40%~7.1%之间(见表4)。说明本方法精密度和准确度良好,对于有机质含量在10%以下的土壤均适用,方法稳定可靠。

**表3:** 低有机质含量土壤中5种化合物的加标回收率和精密度(*n*=6)

Compound	0.02 mg/kg			0.05 mg/kg			0.2 mg/kg	
	Recovery/ %	RSD/ %		Recovery/ %	RSD/ %		Recovery/ %	RSD/ %
AMPA	91.2	0.93		84.7	4.81		86.6	1.4
MPP	97.8	3.4		98.3	3.08		97.5	1.2
GLY	74.6	6.8		74.8	2.02		74.2	4.9
GLUF	101	2.9		98.7	3.07		98.3	2.7
NAG	97.5	6.8		98.8	5.55		94.4	2.6

**表4:** 高有机质含量土壤中5种化合物的加标回收率和精密度(n=6)

Compound	0.02 mg/kg			0.05 mg/kg			0.2 mg/kg	
	Recovery/ %	RSD/ %		Recovery/ %	RSD/ %		Recovery/ %	RSD/ %
AMPA	92.1	1.6		110	1.8		97.5	5.2
MPP	98.6	6.9		115	7.1		113	1.7
GLY	114	3.0		99.1	5.7		90.8	7.0
GLUF	108	3.9		104	6.1		116	0.40
NAG	106	5.7		105	5.0		111	0.51

2.4.3 与其他方法比较

表5对本方法和文献方法进行了比较。本方法中GLY和AMPA的LOD值低于绝大多数文献中报道的非衍生化方法^[7,15,27]^。周芹等^[21]^建立的土壤中GLY和AMPA的非衍生化-UPLC-MS/MS测定方法虽然LOD值低于本方法,但是线性范围的最低浓度为20 μg/L,高于本方法(5 μg/L);而且前处理需要过HLB固相萃取小柱,操作较繁琐,成本高,不适用于大批量土壤样品的快速检测。目前文献中报道的非衍生化方法均未注明所用土壤样品的来源及类型,方法的适用范围不明确。本文建立的方法可以适用于不同有机质含量土壤中GLY、GLUF及其代谢物等5种化合物的同时测定,而且提取液无须过固相萃取小柱净化,无须衍生化处理,操作步骤简单,具有检出限低,准确度高和重复性好等优点。

**表5:** 本文方法与文献方法的比较

Soil source and type	Derivatization	Detection method	Compound	Linear range/ (μg/L)		LOD/ (μg/kg)	Recovery/ %	Reference
Apple field soil (organic matter content 2.65%)	yes	GC-NPD	GLY	100-	20000	20	84.4-94.0	[10]
Sri Lanka farmland	yes	GC-MS	GLY	2-	100	20	83.4	[28]
			AMPA	2-	100	20	87.1	
Mulberry field soil	yes	HPLC-UV	GLY	5000-	35000	-	98.5-103	[11]
Peach field soil (loess soil, black soil and	yes	HPLC-MS/MS	GLY	1-	2000	10	76.7-93.0	[20]
red soil)			AMPA					
Yellow cinnamon soil, lou soil and loess soil	yes	HPLC-MS/MS	GLY	1.6-	200	0.80	84.0-104	[6]
(organic matter content from 0.74%-1.25%)			AMPA	1.6-	200	0.94	84.0-104	
Red loam soil, rice soil and black soil	yes	HPLC-FLD	GLY	10-	500	20	68.3-109	[17]
-	no	IC	GLY	20.0-	1000	40	80.2-86.4	[15]
			GLUF	20.0-	1000	80	87.1-101	
			AMPA	5.0-	400	20	85.8-106	
-	no	UPLC-MS/MS	GLY	20-	200	0.5	79.7-96.2	[21]
			AMPA	20-	200	0.6	77.8-99.5	
-	no	UPLC-MS/MS	GLY	100-	10000	100	85.8-94.6	[27]
-	no	UPLC-MS/MS	GLY	10-	1000	20	77.5-92.0	[7]
			AMPA	10-	1000	20		
			GLUF	5-	500	10		
Peach field soil (organic matter content	no	UPLC-MS/MS	GLY	5-	500	4.0	74.2-78.8	this
2.89%-7.38%)			AMPA				83.7-91.2	method
			GLUF	2-	500	2.0	94.1-101	
			MPP				97.5-104	
			NAG				94.4-108	

NPD: nitrogen-phosphorus detection; -: not indicated in the cited article.

### 2.5 实际样品测定

经前期调研发现,在我国禁用百草枯后,桃园中普遍使用GLY和GLUF除草。采集了20份北京不同桃园的土壤样品(有机质含量在1.47%~8.48%之间),对其GLY、GLUF及其代谢物残留量进行了测定,进一步验证该方法的适用性。所有土壤样品中AMPA、GLY、MPP、GLUF、NAG的检出率分别为45%、25%、10%、5%、5%,GLY及其代谢物检出率均高于GLUF及其代谢物,说明在这些桃园中GLY使用较多。AMPA检出率和含量均高于母体GLY,这是由于AMPA为GLY的主要代谢物,与文献报道相一致^[5,6]^。GLY母体最大残留量为35.2 μg/kg, AMPA最大残留量为147 μg/kg,检出GLY母体的土壤样品都同时检测出其代谢物AMPA。GLY残留量以GLY和AMPA残留量之和计,GLUF残留量以GLUF、MPP和NAG残留量之和计,GLY最大残留量为182 μg/kg,GLUF最大残留量为187 μg/kg。因此,GLY和GLUF的使用在土壤造成的残留需要引起一定的关注。

## 3 结论

本文建立了高效液相色谱-串联质谱法快速同时测定土壤中草甘膦、草铵膦及其代谢物残留量的方法。与文献报道的方法相比,该方法具有简单、快速、前处理无须有机试剂、绿色环保、成本低、准确度和灵敏度高、重现性好等优点,可以满足不同有机质含量大批量土壤样品中草甘膦、草铵膦及其代谢物的快速同时检测,为研究其在土壤中的残留状况和环境行为提供可靠的技术支持。
